# Do It by Yourself: Larval Locomotion in the Black Soldier Fly *Hermetia illucens*, with a Novel “Self-Harvesting” Method to Separate Prepupae

**DOI:** 10.3390/insects13020127

**Published:** 2022-01-25

**Authors:** Daniele Giannetti, Enrico Schifani, Roberto Reggiani, Emanuele Mazzoni, Maria Cristina Reguzzi, Cristina Castracani, Fiorenza A. Spotti, Beatrice Giardina, Alessandra Mori, Donato A. Grasso

**Affiliations:** 1 Department of Chemistry, Life Sciences & Environmental Sustainability, University of Parma, Parco Area delle Scienze, 11/a, 43124 Parma, Italy; enrico.schifani@unipr.it (E.S.); cristina.castracani@unipr.it (C.C.); fiorenzaaugusta.spotti@unipr.it (F.A.S.); beatrice.giardina@studenti.unipr.it (B.G.); alessandra.mori@unipr.it (A.M.); donatoantonio.grasso@unipr.it (D.A.G.); 2 Azienda Agraria Sperimentale Stuard, Strada Madonna dell’Aiuto, 7/a, San Pancrazio, 43126 Parma, Italy; r.reggiani@stuard.it; 3 Department of Sustainable Crop Production, Section Sustainable Crop and Food Protection, Università Cattolica del Sacro Cuore, 29122 Piacenza, Italy; emanuele.mazzoni@unicatt.it (E.M.); cristina.reguzzi@unicatt.it (M.C.R.)

**Keywords:** insects behaviour, insect farming, *Hermetia illucens*, black soldier fly, circular economy, self-harvesting, locomotor activity

## Abstract

**Simple Summary:**

The black soldier fly *Hermetia illucens* is a widespread species of fly of American origins, which is increasingly used to develop sustainable waste recycling processes as it is able to develop by consuming a wide variety of wastes as food, while both its body and the residuals of its feeding activity can be re-used in a variety of processes. However, many aspects of its larval biology remain unknown. Among these, there is larval movement and its variation in response to external stimuli and environmental conditions. Larvae of the black soldier fly eventually reach the prepupal stage, during which they stop feeding and seek a shelter to pupate. Sorting prepupae from the younger larvae and feeding substratum can be important in rearing processes, since they are used to obtain adults but are also particularly rich in protein and lipids. We focused our study on behavioural differences between prepupae and younger larvae, describing tonic immobility as an anti-predatory response of both, but also their very different ways of locomotion and reactions to stress. Finally, we developed a practical system to exploit these differences, inducing prepupae to move away from the substratum and other larvae to be efficiently collected.

**Abstract:**

The neotropical insect *Hermetia illucens* has become a cosmopolite species, and it is considered a highly promising insect in circular and sustainable economic processes. Being able to feed on a wide variety of organic substrates, it represents a source of lipids and proteins for many uses and produces recyclable waste. We investigated the characteristics and differences in the poorly-known locomotory behaviour of larvae of different instars, paying particular attention to the unique characteristics of the prepupal stage, key to farming and industrial processes. Moreover, we attempted to develop a “self-harvesting” system relying on the behavioural traits of prepupae to obtain their separation from younger larvae under rearing condition with minimum effort. Prepupae differ from younger larvae in their response to physical disturbance in the form of tonic immobility and significantly differ in their locomotory movements. Both prepupae and younger larvae reacted similarly to heat or light-induced stress, but low light and high moisture induced only prepupae to migrate away, which resulted in the development of a highly efficient separation methodology. The new data on the behaviour of *H. illucens* not only shed new light on some unexplored aspects of its biology, but also led to develop an inexpensive self-harvesting system that can be implemented in small-scale and industrial farming.

## 1. Introduction

The black soldier fly *Hermetia illucens* (Diptera: Stratiomyidae) is native to the Americas, but today has a cosmopolite distribution due to human activities [1,2,3,4,5]. *Hermetia illucens* is considered one of the key species of great interest developing novel systems to use insects in sustainable practices including agriculture [6,7,8]. In particular, it is considered highly promising to the development of sustainable waste management strategies due to its ability to efficiently consume an extraordinary variety of organic food sources including agro-industrial by-products, e.g., [9,10,11]. In addition to this capacity, *H. illucens* is usable as a protein and lipids-rich feed and food [12,13,14,15] and in the production of bioplastics [16,17,18]. Particularly, the ability to provide proteins and lipids through the larval cycle makes *H. illucens* an extremely valuable tool to achieve pressing sustainability goals [19,20]. Moreover, the larvae processing residues may be utilized in other processes, for instance partly replacing commercial peat in the production of potted plants [21] or as a soil amendment for agricultural production as in the case of byproducts of other biomass processing [22,23].

The development of *H. illucens* larvae may take significantly different amounts of time depending on rearing conditions, and larvae are classified into five to seven instars according to different authors [24,25,26,27]. The last instar larvae, also known as prepupae, are behaviorally unique as they avoid feeding and stay away from light sources, seeking shelter to pupate [28]. Their pigmentation and their reduced mouthparts separate them from the other instars, which are mostly distinguished one from another based on their head size [27]. Optimization of mass-rearing processes is key to the viability of *H. illucens* as an asset for agricultural and industrial waste-recycle activities, and several studies have focused on defining the optimal rearing conditions for larval development and facilitating the adults’ reproduction [29,30,31,32,33,34,35,36,37,38,39,40]. However, another key issue for the rearing processes can be the separation of the prepupae from the substrate and the younger larvae prepupae can be kept separate from younger larvae to obtain reproducers. Furthermore, it can be used as feed or even to produce bioplastics due to their significant lipid and protein content [13,16,41,42,43,44]. While prepupae are easily distinguished by their dark pigmentation compared to younger larvae, separating them from the substratum and from the younger larvae may require hand-picking, which is time-consuming, or sifting, whose effectiveness is strongly dependent on the substratum. Investigation on separation methods so far remained scarce despite the key importance of this process. Sheppard and Newton [45] offered a rare exception, describing a way to induce prepupae to “self-harvest” by exploiting their instinct to migrate away from the food source they fed upon as larvae. Unfortunately, they did not quantify the efficiency of their methodology: for example, they did not specify the number of prepupae they were able to collect with their technique compared to those that did not “self-harvest”, and neither did they quantify the frequency of younger instars ending up in the same collection area with the prepupae.

Despite the great interest in this insect, many basic aspects concerning *H. illucens* larvae remain so far uninvestigated, one of which is the description of their locomotory movement. Indeed, the locomotion and related behavior of fly larvae are only rarely subject to focused descriptive investigations [46,47,48]. Moreover, in the case of *H. illucens*, it is notable how little is known of this widely distributed and heavily studied species apart from what concerns topics of very direct relevance with laboratory farming and possible applications.

We investigated larval locomotion in *H. illucens*, documenting the behavioral responses of the apod larvae belonging to different instars to both mechanical disturbance, and stress caused by different light or heat conditions. We compared the prepupae’s locomotory behaviour with the younger instars, which is known to differ mainly since prepupae do not feed and are devoted to finding a shelter to pupate (migration). Exploiting these differences, we attempted to develop and quantify the efficiency of a method to induce prepupae to separate from the younger larvae, resulting in a “self-harvesting” strategy useful under rearing conditions.

## 2. Materials and Methods

### 2.1. Housing

*Hermetia illucens* larvae were bred at the “Azienda Agraria Sperimentale Stuard” (Parma, Italy) farm from a colony regularly bred in the lab of the Department of Sustainable Crop Production, Section Sustainable Crop and Food Protection, Università Cattolica del Sacro Cuore (Piacenza, Italy). They were kept in plastic boxes (60 × 40 × 20 cm) under the following conditions: T: 29 ± 1 °C, RH: 60 ± 0.5%, photoperiod 12:12 (L:D), 4–5 larvae per cm^2^. To feed the larvae, we used 1 kg of organic material daily (corresponding to about 100 mg per larva every day): this consisted in 500 g of pumpkin (*Cucurbita moschata* Duchesne ex Poir.) and 500 g of vegetal wastes: according to their availability in the farm, lettuce (*Lactuca* sp.), summer squash (*Cucurbita pepo* L.), spinach (*Spinacia oleracea* L.), radish (*Raphanus raphanistrum* subsp. *sativus* (L.) Domin), common chicory (*Cichorium intybus* L.), beet (*Beta vulgaris* L.), parsnip (*Pastinaca sativa* L.), rhubarb (*Rheum* × *hybridum* Murray), and tomato (*Solanum lycopersicum* L.).

### 2.2. Larval Instar Classification

**Figure 1:**
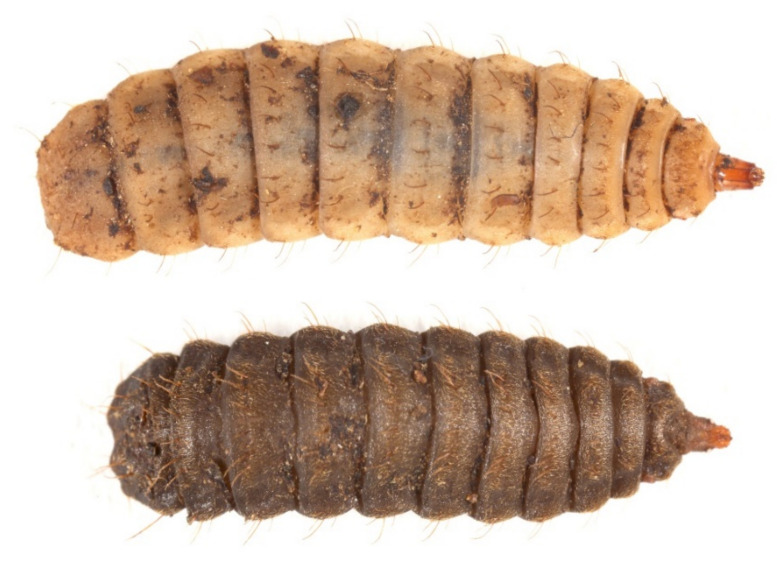
Larval instar classification; on the above the larval instar 6 and below prepupae. Scale bar: 1 mm.

We followed the latest classification (proposed by Glicorescu et al. [27]), which divides larval development into seven instars. According to this classification, the seventh larval instar corresponds to what many other authors have described as prepupae, and we retain the use of this term in this paper. The prepupae and the 6th instar larvae, which are the main targets of our study, are easily identifiable without any harm to the insect and were therefore always identified (Figure 1). Prepupae differ from all other larval instars due to their dark pigmentation in addition to reduced mouthparts, while 6th instar larvae are distinguishable from the younger instars due to their head capsule width above 0.9 mm [27]. On the other hand, younger instars were classified into size-based groups due to the “difficulty of achieving a precise, but harmless, instar-level” classification of living individuals.

### 2.3. Laboratory Conditions

All laboratory experiments were conducted from March to June 2021 under the following environmental conditions: T 26 °C, RH 50% ± 0.5%.

### 2.4. Larval Response upon Disturbance

We analysed larval reaction to mechanical disturbance and its differences according to instars size. We created seven groups of larvae of different sizes, each composed of 100 individuals (min-max values in mm): A: 4–6; B: 7–9; C: 10–12; D: 13–15; E: 16–18; F: 18–24; G: 13–21). The groups F and G are entirely constituted by 6th instar larvae and prepupae respectively. Each larva was tested in a 5 × 10 cm arena which it was free to explore. An entomological forceps was used to gently poke each crawling larvae on their sixth tergite, after which we recorded the following data: (i) whether the larvae stopped moving or not; (ii) for those larvae who became immobile, how much time they remained still.

### 2.5. Locomotory Pattern

We attempted to determine the differences in the locomotory patterns adopted by 6th instar larvae and prepupae. We recorded 4-min HD videos of crawling larvae (6th instar and prepupae, n = 10 for each group) filmed from a lateral perspective on a plastic arena using a 6D camera (Canon, Tokyo, Japan and a Canon EF 100 mm f/2.8 L Macro ISUSM lens. These videos were then analyzed using the trajectory-tracking function of the software Kinovea 0.9.5 (www.kinovea.org, accessed on 16 December 2021) by keeping track of the movements of the insects’ 2nd segment: the output was a database containing the horizontal and vertical coordinates of the sternite during each frame [49].

Single images were extracted from the videos, showing three moments when similar key movements were performed by 6th instar larvae and prepupae. Two representative segments of the cephalic and caudal areas which are involved in these movements were selected in order to compare their differences by using the software ImageJ [50]. Accordingly, the following measurements were taken: (i) when the larva stretches its head forward, before touching the substratum, we calculated the ratio between the maximum height from the substratum of the 9th sternite (length between the sternite and the substratum) and the thickness of the corresponding 9th body segment (length between the sternite and the tergite); (ii) when the head then touches the substratum, before being used as a pin to crawl forward, we calculated the same index, but this time based on the 3rd sternite and 3rd body segment instead of the 9th; (iii) when the head is used as a pin to crawl forward, we calculated the angle it formed with the rest of the body.

### 2.6. Locomotory Speed under Different Stress Conditions

We attempted to verify which environmental conditions could affect the crawling speed of larvae and the possible differences between 6th instar larvae and prepupae. We considered increased heat (compared to the rearing conditions) and light exposure (considering that larvae are lucifugous) as stressful conditions to test. The experimental apparatus consisted in a plastic arena (17 × 6 cm), divided in an open portion directly exposed to light and heating (13 × 6 cm) and a portion covered by a small plastic roof (4 × 6 cm). Larvae were introduced to the open part of the arena, and we recorded HD videos of their behavior; they were placed next to the center of the 6 cm side, opposite to the plastic roof, with their bodies directed towards the latter so that they were induced to move in that direction; then we evaluated their movement speed by recording the time they spent to travel for 13 cm (the length of the heated part of the arena, until they reached the portion covered by a plastic roof). To set different combinations of light and heat conditions, we employed a heated mat (14 W, positioned below the arena for a length of 13 cm, maintaining a temperature of 32 °C) as well as lamps positioned 30 cm above the arena. The characteristics and effects of these lamps were measured using a CRI illuminance meter (CL-70F, Konica Minolta, Tokyo, Japan) to characterize the light spectrum, a luxometer (Hd 8366, Delta Ohm, Padova, Italy) to quantify the lux on the apparatus, and a laser thermometer (Universal Temp, Bosch, Gerlingen-Schillerhöhe, Germany) to quantify the illuminance and temperature (calculated as a mean of two measures taken at 3.5 and 7.5 cm height on the apparatus in order to check the uniformity of the temperature). The following lamps with the following characteristics were tested during the experiments: a warm white LED (150 W, 2452 lumen, Tcp 2582 K, wave length peak 610 nm, 2000 lux, (Incaled, Philips, Amsterdam, Netherlands); a cold white LED (150 W, 2452 lumen, Tcp 3914 K, wave length peak 595 nm, 2000 lux, Philips Incaled); an infrared lamp (150 W, wave length peak 780 nm, 7000 lux, Philips infrared). In addition, tests were also conducted under the ambient light conditions of the laboratory (210 lux) or under conditions of darkness thanks to night vision device, (Night vision monocular 1.2 × 50, Bresser, Rhede, Germany). As a result, the following 7 treatments were created (temperature values measured on the substratum in parentheses):

1. No light (24 °C)
2. No light + heated mat (32 °C)
3. Ambient light (24 °C)
4. Ambient light + heated mat (32 °C)
5. Cold white light (24 °C)
6. Warm white light (32 °C)
7. Infrared light (33 °C)

Each treatment was paired with the presence or absence of a substratum consisting in residual waste produced by the larvae feeding activity (substrate RH 8.5–10.3%, maintained at 24 °C for 48 h). A total of 40 individuals from the 6th instar and 40 prepupae were tested under each possible combination of heat and light treatments and of substratum conditions (n = 7 × 2 × 40 = 560 for each of the two larval instars tested).

### 2.7. Separation of Prepupae from the Younger Larvae

**Figure 2:**
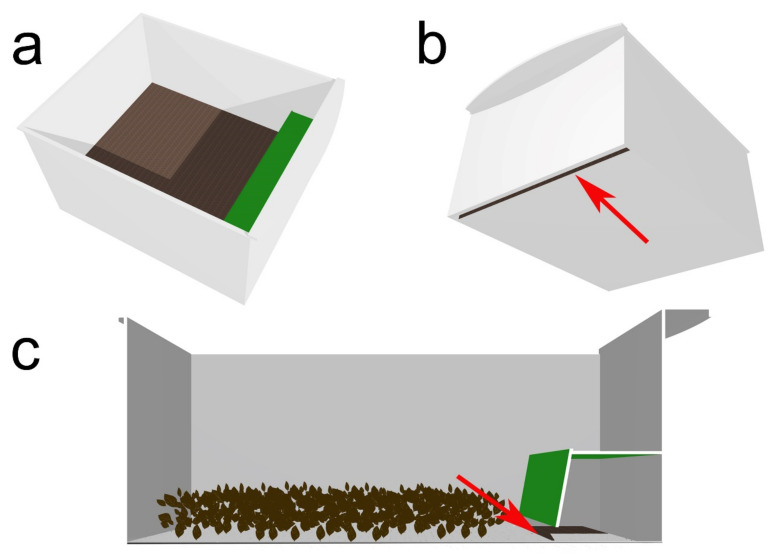
Experimental apparatus to separate prepupae from younger instars. (**a**) View from above. (**b**) View of the underside, the red arrow show the passage for larve (**c**) lateral section view, as shown above the red arrow indicates the 1 cm-wide passage at the base of the barrier through which the larvae can reach the collecting arena.

Based on the results of the previous experiments, we attempted to build an apparatus capable of inducing prepupae to separate from younger instars (self-harvesting). This consisted in a plastic box (29 × 35 × 17 cm) in which a covered area was added. The covered area was placed near one of the short sides of the box, characterized by the presence of a plastic barrier obscuring light. The barrier consisted in a roof (6 cm tall) and a lateral semi-wall allowing the larvae to enter through a narrow passage at its base (1 cm wide) which was left opened towards the non-covered side of the box (Figure 2). Larvae entering the covered area would then find a transversal slit on the floor adjacent to the side of the box (1 × 29 cm), through which they could fall into a collecting arena placed below. In each experiment, we introduced into the open area 300 g of substratum (residual waste produced by the larvae feeding activity, maintained at 24 °C for 48 h), as well as 50 prepupae, 50 larvae of the 6th instar and 50 larvae of younger instars. Experiments lasted 45 min after the insertion of the larvae and prepupae into the boxes; at the end, we counted the larvae and prepupae found in the collecting area. The experiments were carried on exposing the larvae and prepupae to three different conditions of moisture of substratum composed by residual waste: low moisture (no water added to the substratum, RH: 10–11%), medium moisture (6 mL of water per 40 cm^2^ of substratum, RH: 22–23%) and high moisture (13 mL of water per 40 cm^2^ of substratum, RH: 33–34%). Moreover, five light conditions were obtained through the use of commercially available lamps or ambient light as in the previous experiment (lux measurement with a Delta Ohm Hd 8366 luxometer). Here we only focused on ambient light, as the simplest solution achievable, and infrared light as the better-performing based on the locomotory speed experiment, and therefore we utilized the following treatments: 6 lux, 12 lux, or 60 lux (ambient light reduced by a filter), 210 lux (ambient light), >7000 lux (infrared light obtained with a lamp). A total of 10 replicas was conducted for each of the possible combinations of moisture and light conditions (n = 3 × 5 × 10 = 150).

### 2.8. Statistical Analyses

Statistical analyses were performed by using the softwares SPSS 27 (IBM Inc., Armonk, NY, USA) and R 4.0.3 (R Core Team 2021, Vienna, Austria) [51]. Mann Whitney U tests were used in the analysis of differences in the locomotory pattern of larvae belonging to different instars (6th instar vs. prepupae). Kruskal-Wallis tests, followed by pairwise comparisons, were utilized to evaluate differences in the locomotory speed under different treatments. Finally, a general linear model was run to analyse the efficiency of the separation process between prepupae and younger instars according to different treatments.

## 3. Results

### 3.1. Larval Response upon Disturbance

Larvae of group G (prepupae) were the only instar to consistently respond to disturbance by immobilizing their body, 76% of the recorded responses, (see Supplementary Material Video S1), while all the younger larvae (groups A to F) did so only very rarely (1–5%). Duration of the immobility for prepupae was in average 19.72 s (±0.42 SD).

### 3.2. Locomotory Pattern

Prepupae and 6th instar larvae remarkably differed in their locomotory patterns (see Supplementary Material Video S2). Yet, some elements were common to both: they inarched the middle section of their body while stretching their head forward, then approached the substratum with the head while lifting their anterior segments, and, finally, they forcefully utilized their head as a pin to crawl forward.

**Figure 3:**
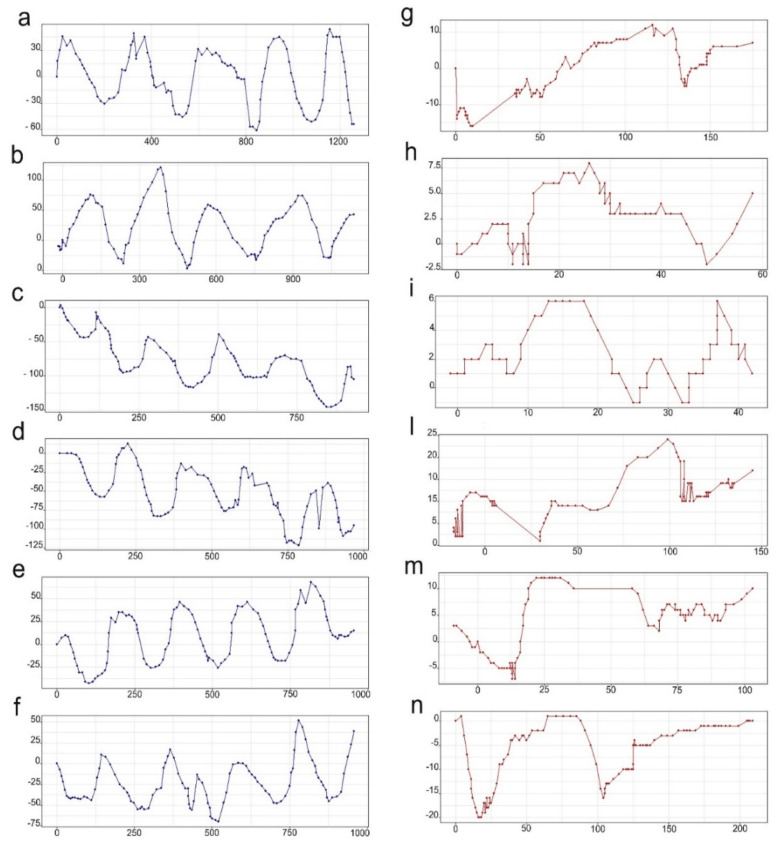
Locomotory patterns of 6th instar larvae (**a**–**f**) and prepupae (**g**–**n**) as described by the trajectory-tracking function of the software Kinovea. The X-axis and Y-axis indicate the horizontal and vertical components of the registered respectively movements.

Prepupae crawled forward by performing a relatively quick and continuous sinusoidal movement of their body, while 6th instar larvae relied more heavily on pinning with their head to move forward. This fundamental difference was well-described by Kinovea trajectory-tracking feature (Figure 3).

**Figure 4:**
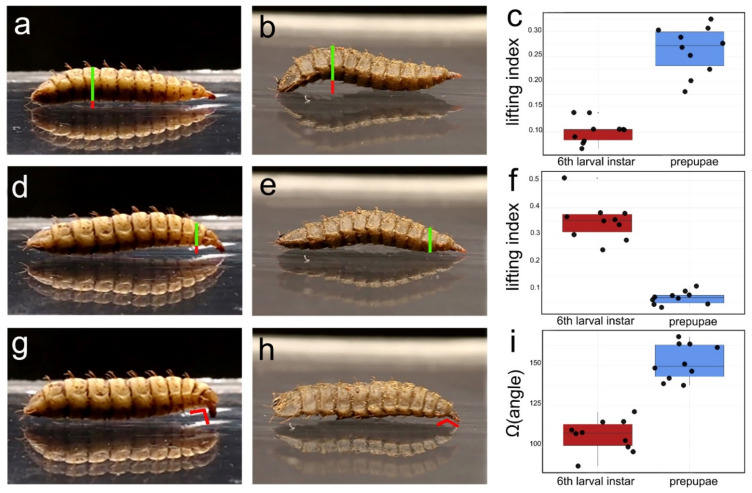
Movements in the locomotory pattern of 6th instar larvae (**a**,**d**,**g**; n = 10) and prepupae (**b**,**e**,**h**; n = 10) and analysis of their differences through measurements (**c**,**f**,**i**). Each row corresponds to one of the three movements: i = when the larva stretched its head forward (**a**,**b**), ii *=* when the head then touched the surface before being used as a pin to crawl forward (**d**,**e**); iii *=* when the head is used as a pin to crawl forward (**g**,**h**). In pictures (**a**–**e**) the red line and green lines indicate the measured characters (the length of the red line is divided for the length of the green one to compute the lifting index), in pictures (**g**,**h**) the measured angle is indicated in red.

Highly significant differences were found when comparing the three selected traits of larval movement: middle body arching was much more emphasized by prepupae compared to 6th instar larvae (U = 100; z = 3.7; *p* < 0.001), the arching of the anterior part preceding the pinning movement was more evident in 6th instar larvae (U = 0.00, z = −3.7, *p* < 0.001) and the angle formed during the pinning movement was also much more acute in the latter (U = 0.00, z = −3.7, *p* < 0.001) (Figure 4).

### 3.3. Locomotory Speed under Different Stress Conditions

**Figure 5:**
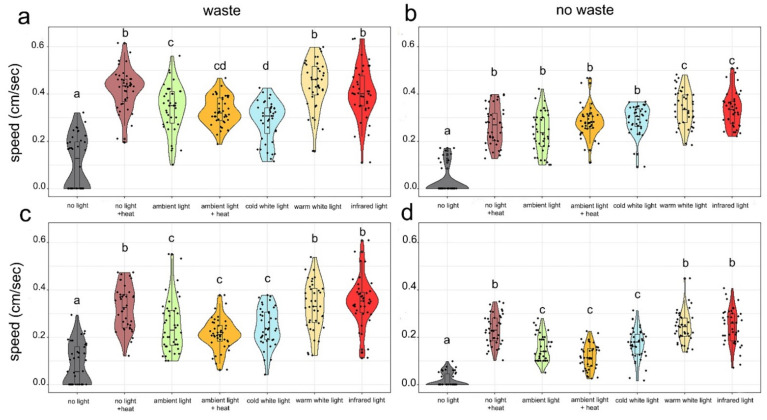
Locomotory speed of 6th instar larvae (above: **a**,**b**) and prepupae (below: **c**,**d**) under different stress conditions. Different letters highlight statistically different groups according to pairwise comparisons. Violins show the probability density of the data at different values.

We detected significant differences in the speed of larval locomotion depending to the different light and heat conditions that were tested, for: 6th instar larvae with waste as a substratum (H_(6)_ = 130.69, *p* < 0.001), 6th instar larvae without waste as a substratum (H_(6)_ = 141.68, *p* < 0.001), prepupae with waste as a substratum (H_(6)_ = 168.19, *p* < 0.001) and prepupae without waste as a substratum (H_(6)_ = 123.71, *p* < 0.001). The waste substratum resulted in an averagely slower movement locomotory speed (Figure 5). Pairwise comparisons revealed that a quicker locomotion was always performed under warm white light and infrared light treatments. In addition, the No light + heat treatment was not significantly different from the aforementioned two in all cases except for that of 6th instar larvae with waste substratum. Conversely, larval locomotion was very slow in the absence of both light and heat (no light treatment).

### 3.4. Separation of Prepupae from the Younger Larvae

**Figure 6:**
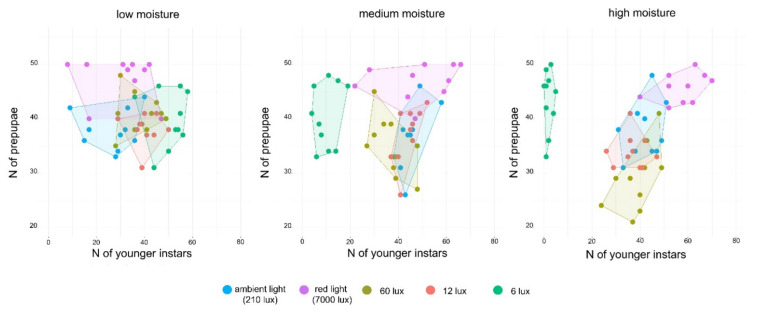
Scatter plots describing the number of prepupae (y axis) and younger larvae (x axis) found in in the collecting arena following separation experiments conducted at different moisture conditions and under different light treatments. A higher efficiency is reached near the upper-left corner of the graph, indicating a high number of prepupae but also a low number of younger instars at the same time.

Different treatments resulted in significantly different outcomes in respect to the number of prepupae and younger larvae found in the collecting area at the end of the experiments. Interestingly, differences between light treatments were more marked when moisture was higher. An optimal result was achieved with the 6 lux light treatment under medium but especially under high moisture conditions: both were detected as highly significantly different treatments compared to the others (*p* < 0.001) and were characterized by a large number of prepupae accompanied by just a very small number of younger instars reaching the collecting area. Expressed as mean ± SD on a percentage basis, this was 87 ± 11% prepupae vs. 4 ± 3% younger instars reaching the collecting area for the high moisture treatment, and 81 ± 12% prepupae vs. 20 ± 9% for the medium moisture treatment (Figure 6) (see Supplementary Material Video S3).

## 4. Discussion

Our study focused on overlooked behavioural aspects of an otherwise intensively investigated insect of increasing importance worldwide, revealing a number of previously unknown patterns.

Our data suggest that tonic immobility, hitherto undescribed in *H. illucens*, to be a standardized response to physical contact in prepupae, while being extremely rare on the younger larvae. Tonic immobility is a defensive anti-predatory mechanism to avoid vibrational and visual detection that can be distinguished from freezing by occurring only after direct contact between the predator and its prey [52,53]. It may sometimes coincide with the concept of thanatosis, but that depends on the possibility that the interacting predator actually “believes” or acts as if the prey is dead [53]. The reason why this behaviour is comparatively so rare in younger larvae of *H. illucens* is difficult to interpret due to the lack of data concerning the natural potential threats that the species faces during its pre-immaginal development. However, it is notable that prepupae couple this behavioural adaptation with a dark-pigmentation: the latter may act as an effective cryptic camouflage by reducing chances of detection by visual predators, especially during tonic immobility. There is currently no data regarding the eventual use by the larvae of chemical cues during locomotion for aggregation, foraging and/or defense as in other (solitary or group living) insects [54,55,56,57,58].

The larval locomotion of *H. illucens*, here described for the first time, strongly differs between younger larvae and prepupae, with the latter lacking a strong role of the head movement that characterizes the former but also the larvae of other Brachycera [48]. This difference may lay in the modification of the mouthparts during the transition between the 6th instar and the prepupae: the first have functional mouthparts, which are severely reduced in the latter [26,27]. To the best of our knowledge, ours is the first use of the software Kinovea to describe the movement patterns of an insect, while it is employed in other different research contexts [59,60,61,62], encouraging further applications to study the movements of other animal species [63,64,65,66,67].

When exposed to stress conditions provided by different light and temperature conditions, 6th instar larvae and prepupae responded similarly. Interestingly, both were able to move quicker in the rather artificial condition of a plastic substratum than in the presence of residual waste, and both had their lowest movement speed under complete darkness and no heating. The latter outcome was an expected result, as the combination of darkness and ambient temperature was supposed to represent a “no-stress” control treatment for *H. illucens* larvae: they are thought to spend their life in a lightless environment within rotten material and soil, and excessive heating normally stimulates dipteran larvae to move quicker in search for more favourable conditions (e.g., [68]). However, it was interesting and surprising to observe that the highest movement speed was reached not only in the presence of strong illumination and heating, but also as a response to the combination of heating and darkness. On the other hand, treatments consisting in heating and intermediate light conditions resulted in more intermediate locomotory speed.

In the separation experiments, coherently with the abovementioned results, we found that the highest numbers of larvae left the boxes and reached the collecting arena whenever illumination was stronger (infrared light). However, this proved to be an ineffective tool to elicit separation of prepupae from the younger instars, since all larvae responded similarly independently from their instar–causing a mix of prepupae and younger larvae to occur in the collecting arenas. On the contrary, the most efficient treatment consisted in the lowest illumination: this was enough to stimulate the prepupae to leave but did not have the same effect on the younger instars, so that almost exclusively prepupae were found in the collecting arenas. These data suggest that prepupae, likely driven by the instinct of locating an adequately safe area to pupate, are more sensible to slight light variations than younger instars, which seem to tolerate a low illumination. Moreover, moisture also played a key role and highlighted another major behavioural difference between prepupae and younger instars: while unaffected by low illumination, younger instars would move away from the substratum if the latter was too dry. Their affinity to higher moisture conditions may perhaps be caused by a need for wet decaying food or a higher vulnerability to water loss through evaporation. However, whatever the reason, this characteristic made it necessary to combine at the same time low illumination with high moisture of the substratum in order to obtain an efficient separation of prepupae from both the substratum and the younger instars.

## 5. Conclusions

In conclusion, we were able to start defining some key aspects of larval locomotion in *H. illucens*, which, despite the wide literature on this insect, remained hitherto unexplored. Further assessment on the behavioural responses of black soldier fly larvae will greatly benefit from investigations targeting the life cycle of wild populations, which is still little-known. However, improving the understanding of basic behavioral aspects of these insects proved immediately promising for applicative uses as well. Our results encourage the development and refinement of insect farming systems based on behavioral data, and particularly of self-harvesting methodologies able to exploit insects’ behavioral attitudes to reduce the need for direct human intervention in the rearing processes. The method we developed, which requires very unexpensive tools and no consume of electric energy to be replicated, proved to be highly efficient in its aim to separate *H. illucens* prepupae from younger instars, and is the first method with such purpose to be tested and described in detail in the publically available scientific literature. As the separation of these different developmental stages appears key to current and future applications of the black soldier fly, it could be easily adopted to optimize small-scale and industrial farming alike with minimal costs.

## Data Availability

Data is contained within the article or supplementary material.

## Supplementary Materials

The following supporting information can be downloaded at: https://www.mdpi.com/article/10.3390/insects13020127/s1, Video S1: Disturbance test; Video S2: Locomotory patterns; Video S3: Separation of prepupae from the younger larvae and the substratum.
